# Detection of novel astroviruses MLB1 and MLB2 in the sera of febrile Tanzanian children

**DOI:** 10.1038/s41426-018-0025-1

**Published:** 2018-03-14

**Authors:** Samuel Cordey, Mary-Anne Hartley, Kristina Keitel, Florian Laubscher, Francisco Brito, Thomas Junier, Frank Kagoro, Josephine Samaka, John Masimba, Zamzam Said, Hosiana Temba, Tarsis Mlaganile, Mylène Docquier, Jacques Fellay, Laurent Kaiser, Valérie D’Acremont

**Affiliations:** 1 Laboratory of Virology, Infectious Diseases Service, University Hospitals of Geneva, Geneva, 1211 Switzerland; 2 University of Geneva Medical School, Geneva, 1211 Switzerland; 3 Department of Ambulatory Care and Community Medicine, University of Lausanne, Lausanne, 1011 Switzerland; 4 Swiss Tropical and Public Health Institute, University of Basel, Basel, 4002 Switzerland; 5 Swiss Institute of Bioinformatics, Geneva, 1211 Switzerland; 6 Global Health Institute, School of Life Sciences, Ecole Polytechnique Federale de Lausanne, Lausanne, 1015 Switzerland; 7 Swiss Institute of Bioinformatics, Lausanne, 1015 Switzerland; 8 Ifakara Health Institute, Dar es Salaam, United Republic of Tanzania; 9 Amana Hospital, Dar es Salaam, United Republic of Tanzania; 10 iGE3 Genomics Platform, University of Geneva, Geneva, 1211 Switzerland; 11 Precision Medicine Unit, Lausanne University Hospital, Lausanne, 1011 Switzerland

Fever is responsible for up to 80% of all pediatric (<5 years of age) outpatient visits in Sub-Saharan Africa. Even in highly endemic areas, malaria rarely causes more than 30% of cases^1^. The remaining episodes are presumably of viral origin but are rarely documented as such. Recent outbreaks due to the Zika, Chikungunya, and Yellow fever viruses have provided an urgent incentive to better identify these fevers’ etiologies. Unbiased high-throughput screening has allowed the discovery of unexpected viral infections, such as those not previously considered to be human pathogens or strains that are too genetically divergent to be recognized by conventional targeted screening methods. These advances have also identified the importance of “commensal” organisms, including viruses, that may significantly impact health and disease. Next-generation sequencing (NGS) is one of the most powerful tools in this new wave of pathogen discovery, providing high-resolution nuances to human pathogenomic diversity.

Using this approach in pediatric samples from an outpatient clinic in urban Tanzania, we describe the presence of the novel human astroviruses, (HAstV)-MLB1 and MLB2, in two patients with different clinical outcomes.

HAstV-MLB1 and MLB2 were discovered in the late 2000s from human stool samples and are considered to be novel HAstV as they are highly divergent from classical HAstV (serotypes HAstV-1 to 8)^2^. In contrast to classical HAstV, a major cause of gastroenteritis^3,4^, the disease spectrum associated with these novel HAstV remains to be characterized^5,6^. Recent findings suggest that susceptible populations to novel HAstV complications include immunocompromised and pediatric ≤ 4-years patients^7^. However, without routine screening, definitive conclusions remain impossible. A previous seroprevalence study revealed a particularly high occurrence of HAstV-MLB1 among children, suggesting that primary infection occurs during childhood^8^. Both HAstV-MLB1 and MLB2 have rarely been detected outside the gastrointestinal tract. To date, HAstV-MLB2 infection has been found in plasma and nasopharyngeal swabs from two febrile children^9,10^ and in the cerebrospinal fluid of two adult patients (one of whom was immunocompromised)^11^. By contrast, HAstV-MLB1 has only been reported outside the gastrointestinal tract in a 4-year-old immunocompromised patient with disseminated infection^12^.

Here, we report the results of two novel HAstV infection cases in individuals who were part of a large pediatric cohort (2 months–5 years of age) recruited at nine outpatient clinics in Dar es Salaam, Tanzania. Unbiased NGS was performed on 135 serum samples collected between December 2014 and February 2016. The samples were selected randomly from a cohort presenting with either “fever without a clinical focus” or “severe febrile disease requiring hospital referral”. Participants in this study were malaria-negative by rapid diagnostic testing and were managed by electronic clinical algorithms^13^. RNA and DNA libraries were prepared as previously described^14^ for analysis by NGS paired-end sequencing using the 100-bp protocol. The HiSeq 2500 and 4000 platforms (Illumina, San Diego, US) were used for RNA and DNA, respectively, generating an average of 96 million reads per sample. Raw data were analyzed using an updated version of the ezVIR pipeline^14^.

Novel HAstV were detected in two patients (raw data available here: 10.5281/zenodo.1094970), Case #1 (158 HAstV-MLB1 reads, genome coverage = 29.54%) and #2 (459 HAstV-MLB2 reads, genome coverage = 95.83%) (Fig. 1 and Supplementary Figure S1). Each case’s clinical presentation is summarized below.

**Fig. 1:**
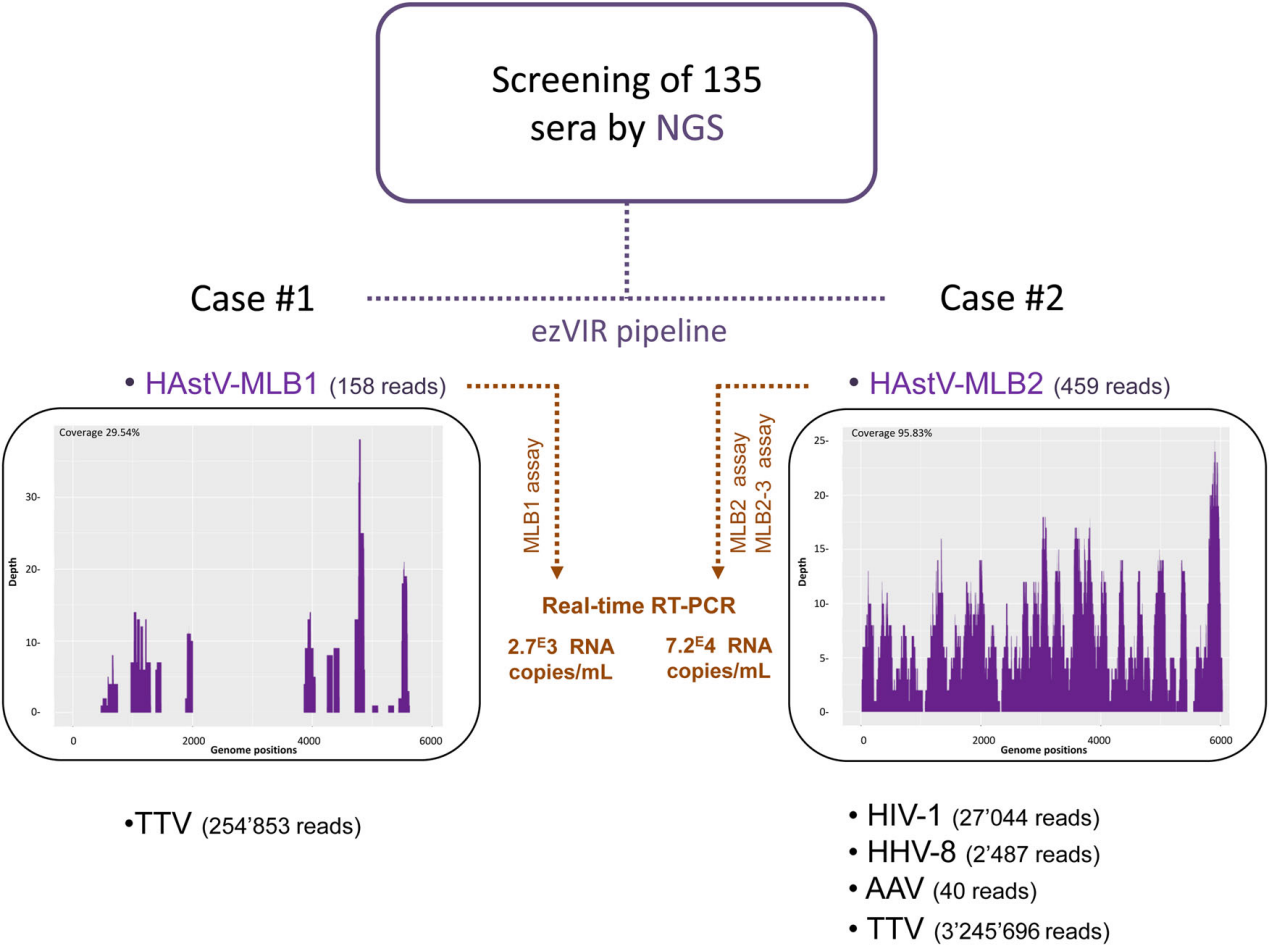
Sample analysis flowchart. Purple: NGS analyses. Orange: specific HAstV-MLB1 and HAstV-MLB2/MLB2-3 real-time RT-PCR confirmation assays. The number of reads for each specific virus detected by ezVIR is indicated. Read coverage histograms are shown for HAstV-MLB1 and HAstV-MLB2. *NGS* next-generation sequencing, *TTV* torque teno virus, *HIV-1* human immunodeficiency virus type 1, *HHV-8* human herpesvirus 8, *AAV* adeno-associated virus

Case #1: a 9-month-old male presented with a 3-day history of fever (axillary temperature of 37.6 °C) and no other complaints or signs of focal infection. Malaria rapid diagnostic testing and blood cultures were negative; the urine dipstick was normal. The patient had no underlying co-morbidities reported and tested negative for HIV at inclusion. Additionally, his mother reported an HIV-negative result during routine prenatal screening. A diagnosis of “presumed viral infection of unknown etiology” was assigned. The patient did not receive any antimicrobials and was asymptomatic by day 3 of follow-up.

In addition to the aforementioned HAstV, NGS also detected a torque teno virus (TTV, 254 853 reads) (Fig. 1 and Supplementary Figure S1). HAstV-MLB1 presence was confirmed by real-time RT-PCR with a viral load estimated at 2700 RNA copies/mL per the MLB1 assay previously described^7^.

Case #2: a 3.5-year-old male presented with a 5-day history of fever and cough. A past medical history of untreated HIV infection and chronic malnutrition were also documented. A temperature of 38.9 °C was measured at triage accompanied by tachypnea but with normal oxygen saturation (respiratory rate: 60 bpm, O_2_Sat: 99%). Laboratory workup showed severe anemia (Hb 3.2 gm/dl), and HIV infection was confirmed by rapid test; however, no CD4 + T-cell counts were available. Malaria RDT and blood cultures were negative, and the urine dipstick was normal. C-reactive protein was measured at 80 mg/dL; lactate was 3.5 mmol/L.

An initial diagnosis of “sepsis and severe anemia” was assigned, and the patient was immediately hospitalized. The hospital-based treatment included packed red blood cells and presumptive antibiotic treatment with penicillin, gentamicin, rifampicin, and ethambutol (based on clinically suspected tuberculosis). During hospitalization, the child developed progressive respiratory distress with hypoxemia and clinical signs of liver failure with ascites and jaundice. Penicillin/gentamicin was then changed to co-trimoxazole and steroids due to a suspected *Pneumocystis jirovecii* infection (no microbiological confirmation attempted). The patient died on the 8th day of hospitalization. No autopsy was performed. Subsequent NGS analysis on blood samples confirmed the presence of HIV-1 (27,044 reads), HHV-8 (2487 reads), adeno-associated virus (40 reads), and TTV (3,245,696 reads; Fig. 1). HAstV-MLB2 was confirmed by real-time RT-PCR with a viral load estimated at 72,000 RNA copies/mL using the MLB2 and MLB2-3 assays previously described^7,9^. The high viral load may suggest a potential association between HAstV-MLB2 and the illness, especially without an alternative diagnosis and no clinical response to antibiotics.

The presence of a cough in Case #2 is consistent with recent epidemiological data showing that upper respiratory tract manifestations were present in up to 70% of all patients with novel HAstV (albeit in stool samples)^7^. This association is also supported by our recent report on the novel HAstV-VA1 detected in a nasopharyngeal swab of a 13-month-old child suffering from acute respiratory symptoms^15^. Large-scale screening of novel and classical HAstV in routine respiratory samples is needed to test the significance of this possible association. Interestingly, neither Case #1 nor Case #2 presented signs or symptoms of acute intestinal disease, which is known to be associated with classical HAstV.

In conclusion, this study reports the first description (to our knowledge) of HAstV-MLB1 in a blood sample from a patient with transient uncomplicated febrile disease. It is also the first description a HAstV-MLB2/HIV co-infection. The high viral load and fatal outcome in the latter case supports the rationale to investigate the potential pathogenic role of novel HAstV in immunocompromised patients. Therefore, this study underscores that novel HAstV may be implicated in various clinical presentations, and implementing routine molecular assays should be considered in susceptible populations.

## Electronic supplementary material

Supplementary Figure S1

Supplementary Figure ledgend
