# Supplementary figures and images for: Detection of novel astroviruses MLB1 and MLB2 in the sera of febrile Tanzanian children
Source: Emerg Microbes Infect. 2018 Mar 14;7:27. doi: 10.1038/s41426-018-0025-1 (PMC5849711; doi:10.1038/s41426-018-0025-1)

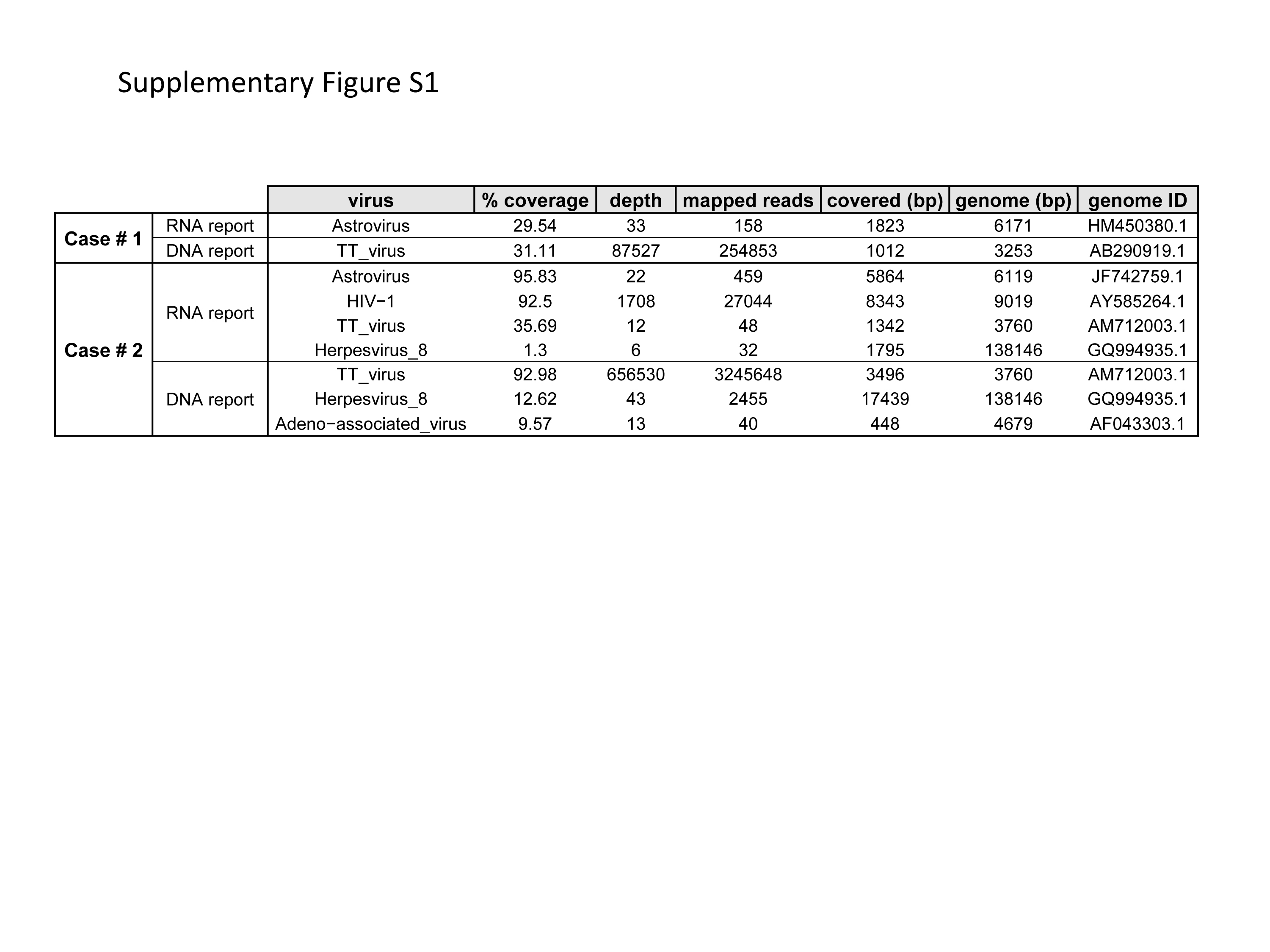

Supplement: Supplementary file 1 — Supplementary Figure S1 [file 41426_2018_25_MOESM1_ESM.jpg]
