# Supplementary material for: Detection of novel astroviruses MLB1 and MLB2 in the sera of febrile Tanzanian children
Source: Emerg Microbes Infect. 2018 Mar 14;7:27. doi: 10.1038/s41426-018-0025-1 (PMC5849711; doi:10.1038/s41426-018-0025-1)
Supplement: Supplementary file 2 — Supplementary Figure ledgend [file 41426_2018_25_MOESM2_ESM.docx]

**Supplementary Figure S1: ezVIR reports for Case #1 and #2.** bp: base pair
